# Connectedness among Urban Parks from the Users’ Perspective: A Systematic Literature Review

**DOI:** 10.3390/ijerph20043652

**Published:** 2023-02-18

**Authors:** Jun Li, Melasutra Md. Dali, Nikmatul Adha Nordin

**Affiliations:** Centre for Sustainable Planning and Real Estate (SUPRE), Faculty of Built Environment, Universiti Malaya, Kuala Lumpur 50603, Malaysia

**Keywords:** connectedness, urban parks, users’ perspective, physical and perceived

## Abstract

Although many benefits of urban green space networks have been consistently demonstrated, most of the discussion on space connectivity has concentrated on ecological aspects, such as patch–corridor–matrix connectivity. There are limited systematic studies that have investigated the connectedness between urban parks and people. This study aimed to explore the connectedness among urban parks from the users’ perspective by using a systematic literature review. By following the PRISMA protocol and analyzing 54 studies from Scopus and Web of Science between 2017 and 2022, we proposed the concepts of physical connectedness and perceived connectedness. The “physical connectedness” contained the dimensions of road attributes and park attributes, as well as six categories including physical accessibility, street connectivity, the street environment, spatial scale, facilities and amenities, and natural elements. The “perceived connectedness” mainly referred to people’s perception of the physical environment. The four categories were perceived accessibility, perceived safety, aesthetics, and Kaplan’s perceptual model. Finally, in terms of individual attributes, the impact of sociodemographic factors (age, gender, income, education, and occupation) and the motivation for activity on park connectedness were also taken into account. On the basis of our findings, this study suggested that park connectedness should not only focus on physical connectedness but also perceived connectedness.

## 1. Introduction

Public green space, as an urban oasis in contact with nature, offers many benefits to the city. It can significantly promote people’s physical health [1,2], reduce psychological stress [3], improve the quality of life [4], and increase a sense of belongingness [5] and social cohesion [6]. These benefits are mostly enjoyed inside the park, while some benefits also expand to the adjacent areas [7], such as reducing the urban heat island effect [8], regulating the air temperature and relative humidity [9], enhancing community connections between people [10,11], and economically elevating property values in the surrounding neighborhood [12].

The urban green space system is a complicated network [13]. On the one hand, the park itself consists of many elements (flora and fauna, water bodies, facilities, and users), and the changes in the combination of its internal elements affect the realization of the value of the park and the visits of residents [14]. Zhang et al. [15] and Dong et al. [16] proposed vegetation as the key quality indicator that affected the park-visiting experience of the residents. Leisure and entertainment facilities also largely determined the frequency of visits to the park [17,18]. Furthermore, the park system can be a green network within the city’s sustainable system, which is dynamically related to other components [14], such as urban functional service facilities, landscape patterns [19], the city’s crime map [20], and the relationship between urban parks and sociodemographic variables [2]. Park [6] mentioned that parks are, to some extent, fragmented and nonconnected and that these green particles should be woven into the broader ecosystem. Menconi et al. [14] declared that one element in a single green space may change the balance of the system. This element can produce positive synergy or conflict with other external elements and variables. Therefore, in order to maximize the value of the green space itself and the extended surrounding benefits, the research about urban parks and public green spaces focuses not only on the individual park but also on the connectedness of parks and the overall park network [21].

The ecological connectivity of green spaces has long been studied, and typically includes both structural and ecological connectivity. Forman [22] proposed the corridor–patch–matrix model, which effectively improved the connectivity of the ecological landscape. High connectivity has been shown to facilitate the flow or movement of ecological processes [23], effectively mitigating the urban heat island effect [24,25] and improving biodiversity [26]. The concept of green infrastructure also emphasizes the interconnection between green infrastructure sites [27]. These studies have mainly focused on the connections and interactions among natural elements such as the location, water bodies, topography, vegetation, animals, and plants, without considering the perspectives of people.

There have been studies on the impact of parks’ connections on park users. Benedict and McMahon [27] mentioned that improvements in walkability between built environments can effectively promote the utilization of proximal public spaces. Street connectivity can affect individual’s physical activity and health [28]. Nursyamsiah and Setiawan [5] noted that residents can indirectly increase spatial connectivity through social coherence. Kovacs-Györi et al. [29] stated that aesthetics and perception can break physical distance thresholds and motivate people to travel. However, there are still issues that have received less attention, such as the different connectedness paths between urban parks, and what factors and indicators of connectedness affect the park’s usage. Based on the research gaps above, the research objective of this study was to explore the connectedness among urban parks from the users’ perspective. Three sub-research objectives (sub-ROs) were as follows:

Sub-RO1. To analyze the characteristics of the geographical distribution, the research methods, and the measurement methods in terms of current research on park connectedness;

Sub-RO2. To develop an emergent framework of park connectedness;

Sub-RO3. To explore the connectedness paths and the relationships of dimension.

This study is divided into six main sections to answer these ROs. This is the introduction, which focuses on the need for the research and the research objectives. The second section defines the meaning of connectedness, and Section 3 explains the systematic literature review method process and the main research phases. This is followed by a review and an analysis of the basic information of the selected articles, such as the geographical distribution, the research method, and the measurement method of connectedness, in Section 4. We then synthesize the research findings into a framework and provide a detailed description of its components. The next section (Section 5) discusses the paths of connectedness, the relationships among the dimensions of the indicators, and future research directions. Finally, the conclusion is given in Section 6.

## 2. The Definition of Connectedness

“Connect” generally has two meanings. One means to gather or contact, to establish a real or notional link; the other is to have a relationship or an intimate relationship with someone [30]. Whatever the meaning, it implies a study of relationships or correlations. In the field of environment research and urban planning, Taylor et al. [31] mentioned that connectivity is described as the extent to which a landscape facilitates or impedes the movement of living things between patches. This is divided into functional connectivity and structural connectivity. Structural connectivity is based on landscape structure; functional connectivity considers the behavioral responses of living organisms to individual landscape elements and to the spatial configuration of the landscape as a whole [31,32]. The connectivity emphasizes the degree to which the physical environment affects living things. The term “connectedness” has often been regarded as an important factor in human development and psychology [33]. Scholars have often described connectedness according to the different dimensions of connectedness. Its dimensions include social connectedness [34,35,36], cultural connectedness [37], and connectedness to nature [37,38]. Hagerty et al. [39] stated that good connectedness is a good psychological feeling when a person actively participates in another object, group, or environment, which emphasizes human participation and perception. In this study, connectedness refers to a bonding process between people and a series of urban parks, taking the various combinatorial connections between parks and people into account. Different connections can be created based on different combinations, for example, between parks, between people and parks, and between people and people.

## 3. Methodology

Our goal was to engage in a comprehensive systematic literature review of connectedness to consolidate the existing work into a meaningful introductory framework of urban park connectedness. We accomplished this goal by adhering to the three phases of article selection, descriptive codification and analysis, classification of the findings, and presentation of the relationships.

### 3.1. Phase 1: Article Selection

**Figure 1:**
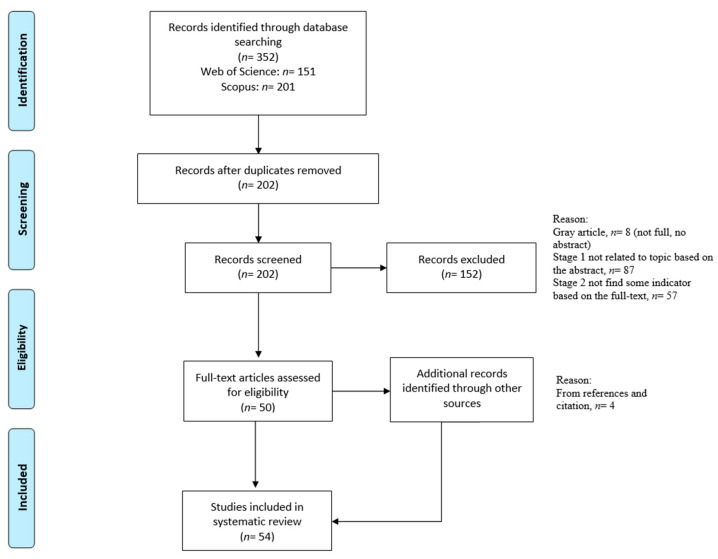
The flowchart of the PRISMA approach used in the systematic literature review study.

In this study, the researchers used the systematic literature review method including the complete PRISMA checklist. A systematic literature review is a method of identifying, evaluating, and interpreting all existing research related to a specific research question, subject area, or phenomenon of interest [40]. The Preferred Reporting Items for Systematic Reviews and Meta-Analyses (PRISMA) was used to establish a systematic literature review (SLR) in this study. The PRISMA procedure included the identification, screening, eligibility and exclusion criteria processes, and the abstraction and analysis of the data, as shown in Figure 1.

#### 3.1.1. Search Strategy

The search was conducted in two academic databases (Scopus and Web of Sciences) in October 2022. To identify the connectedness among urban parks from the users’ perspective, three limitation terms were used in the initial search. The first terms (used as title, abstract, or keyword) were “connectivity” OR “connectedness” OR “continuity” OR “coherence” OR “proximity” OR “accessibility” OR “walkability”. To focus on studies on urban parks, the following search terms were used (as title, abstract, or keyword): “park*” OR “urban park*” OR “public green space*”. From the users’ perspective, the third terms (used as title, abstract, or keyword) were “user” OR “resident*” OR “tourist*” OR “person*” OR “individual” OR “people” OR “human being”. These terms were combined with “AND” to narrow down the search scope. Table 1 lists these terms and synonyms.

#### 3.1.2. Inclusion and Exclusion Criteria

During the screening process, publication between 2017 and 2022 was the first criterion of selection. The second criterion was the article type. Articles, conference articles, reviews, and conference reviews were included. The third criterion for inclusion was that the article had to be written in the English language. According to these three criteria, 352 records were found. After we had eliminated the duplicates, there were 202 records for the subsequent eligibility steps.

In the eligibility process, depending on the research topic and objectives of the article, studies were excluded if they were: (1) on evaluations of ecology or landscape connectivity such as the patch–corridor–matrix models; (2) for special priority groups such as people with disabilities; or (3) occurred in nonurban public green spaces or other green spaces, such as green buffers, urban forests, marshlands, and habitats. These exclusion criteria were set, and the reasons were given, as shown in Table 2. According to these criteria, with an additional four articles from the references and citations, the review eventually obtained 54 selected articles for the systematic literature review.

### 3.2. Phase 2: Descriptive Codification and Analysis

The purpose of the codification phase was to extract the main information of the articles, which include the sociological characteristics of the population, the geographical distribution, the type of green space (parks, road, others), the research methods (qualitative, quantitative, mixed), and the measurement methods (objective measurements or perceived measurements). While reading an article, we extracted this content from each article and summarized it as shown in Supplementary File S1. The trends could be discovered by analyzing the proportions in the results section. An additional outcome of this phase was to explore the indicators of the connectedness among urban parks. In this step, each author independently reviewed the content of the 54 studies and coded each of them. For each article, the key indicator(s), specific measurement standards, and the description of connectedness were identified and briefly summarized based on a comprehensive reading of the articles, with specific attention paid to the abstract, method, and conclusion sections. All the extracted raw data, including the connectedness indicators, are presented in Supplementary File S2.

### 3.3. Phase 3: Classification of the Findings and Presentation of Relationships

Phase 3 involved the process of visualization and induction. There were two steps. First, we visualized the findings on the raw indicators obtained in Phase 2 in order to construct the identified indicators into a series of meaningful categories and dimensions. We then explored the relationships among the dimensions of the indicators.

**Figure 2:**
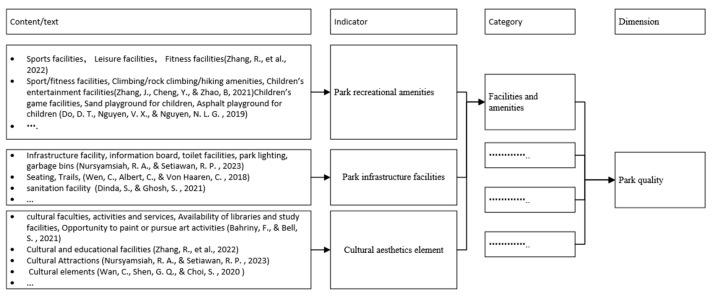
An illustrative example of how the indicators, categories, and dimensions emerged (Wan et al., 2020 [1], Nursyamsiah et al., 2023 [5], Zhang et al., 2022 [15], Zhang et al., 2021 [17], Do et al., 2019 [18], Wen et al., 2018 [41], Dinda et al., 2021 [42], Bahriny et al., 2021 [43]).

The first step A of Phase 3 involved the visualization process of word frequency. Indicators of connectedness in this research were chosen through meticulous manual reading and understanding. Because the specific indicators were usually not the authors’ keywords in their article, software that automatically retrieved keywords, such as VOS viewer or Citespace, could not achieve good results from the analysis. In this study, statistical software for word density, Weiciyun (https://www.weiciyun.com/, accessed on 7 December 2022), was used to calculate the frequency of the indicators by manually importing all indicators. The indicators mentioned repeatedly were used as the indicators of park connectedness in this study, because it is believed that the indicators mentioned and verified repeatedly by scholars are relatively more important. In light of the results of word frequency analysis, the content information that appeared repeatedly was located, and the indicators were summarized after repeated comparisons, and then they were classified into appropriate categories, which were then organized together into abstract aggregate dimensions. For the high-frequency word “facilities”, as an example, this process involved importing the original information of Supplementary File S2 to word frequency analysis, obtaining high-frequency words “facilities”, locating the content information in the raw data, comparing and analyzing all content involved “facilities”, and then summarizing the indicators, categories, and dimensions, as illustrated in Figure 2. This process continued until all the selected indicators had been organized into distinct categories.

As for the second step B, the relationships between the dimensions were further explored in conjunction with the indicators obtained from step A. In other words, we used an inductive approach to conceptualize the relationships among park attributes, road attributes, perception attributes, and individual attributes, to provide a theoretical explanation for connectedness among pocket parks. As we mentioned in the second section, there were different combinations in terms of connectedness, between parks, between people and parks, and between people and people. In response to this, we explored these connections from different perspectives, such as the different connectedness paths, the relationship between physical and perceived connectedness, and the social interaction between people. The iterative and inductive process we followed in steps A and B facilitated the understanding, combination, and reorganization of findings from the indicators of park connectedness literature. Finally, the figure with boxes and arrows in the discussion section illustrated the theoretical relationship between aggregate dimensions and their related people–parks connections.

## 4. Results

### 4.1. Geographical Distribution by Region

Among the samples in the analysis, 70% of the selected studies (*n* = 37) were from Asia, 17% were from Europe (*n* = 9), and 13% were from North America (*n* = 7). The majority of Asian connectedness studies were conducted in China (54%) and Iran (14%). In Europe, most of them were from Romania (33%) and Portugal (22%). In North America, all studies came from the United States of America. Africa, South America, and Australia were not represented at all. Furthermore, in terms of the study city, it can be seen that most of the studies considered the capitals or central developed cities in various countries, such as Beijing, Shanghai, Shenzhen, and Wuhan in China; New York and Las Vegas in the USA; Tehran, the capital of Iran; Bucharest, the capital and commercial center in Romania. Other less central cities were less studied (see Table 3).

### 4.2. Distribution of Articles on the Basis of Research Methods and Measurement Methods

The connectedness among parks can be measured by two methods: objective measurements and perceived measurements. The proportion of objective measurements and perceived measurements was evenly distributed, according to the statistics of the 54 selected articles. Objective measurements such as distance detection, the number of devices, and vegetation structure accounted for 39%; perceived measurements and satisfaction surveys using the Likert scale accounted for 35%; combined objective and perceived measurements accounted for 20%. This proportion shows that both measurement methods are almost equally important and that mixed measurements (objective and perceived) should also be taken seriously.

**Figure 3:**
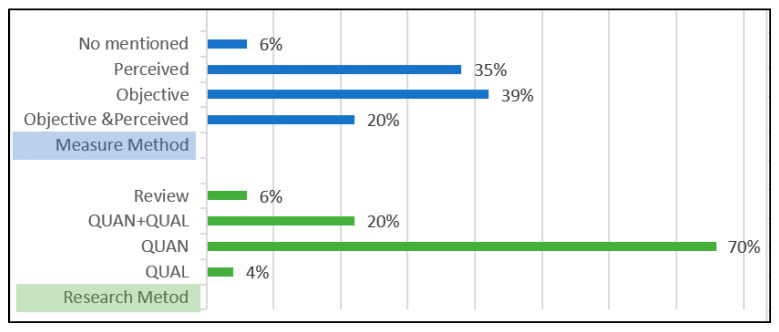
The proportions of different measurement methods and research methods.

Generally, research methods can be classified as quantitative, qualitative, and mixed. Among the 54 articles, about 70% of the studies were quantitative, utilizing questionnaires or surveys. The mixed research method accounted for 20%, while the qualitative method accounted for only 4%, all of which were interviews. This reflects the dominance of quantitative research in the research field of connectedness among urban parks (see Figure 3).

### 4.3. Synthesis of the Findings Regarding Indicators

According to the method mentioned in Step A in Section 3.3, this section explains the results of visualization analysis and describes the detailed components of the connectedness indicators framework through word frequency density as well as comparison and inductions.

#### 4.3.1. Word Frequency Density Analysis of the Indicators

**Figure 4:**
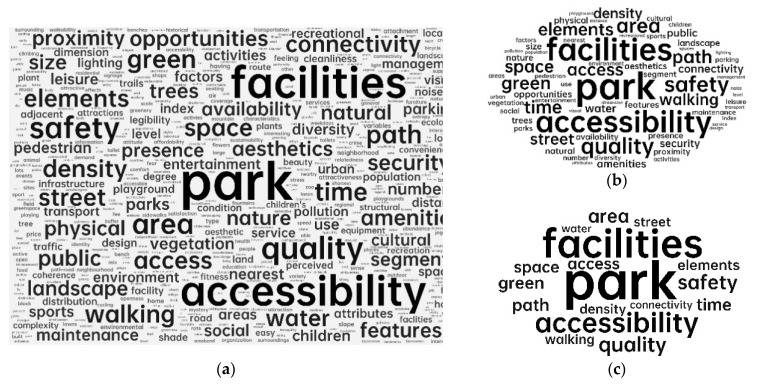
Visualization of the word frequency analysis: (**a**) word frequency of all indicators; (**b**) word frequency of indicators appearing more than 5 times; (**c**) word frequency of indicators appearing more than 10 times.

**Figure 5:**
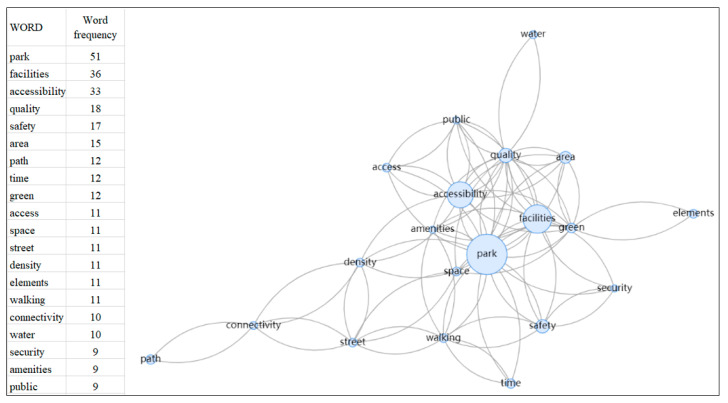
Relationship analysis of the top 20 words of all indicators.

The study was visualized for the raw word frequency of all indicators; the result is shown in Figure 4. Figure 4a shows the word frequency density of all indicators, Figure 4b shows the frequency of words that appearing more than five times, and Figure 4c shows the frequency of words appearing more than ten times. The larger the size of the word, the greater the frequency of its occurrence. From this analysis, we can see that some indicators, such as facilities, accessibility, safety, quality, park area, street, walking, and paths, were mentioned repeatedly. In addition, the relationships of the top 20 words are visualized in Figure 5. There were strong correlations among parks, facilities, accessibility, quality, and safety. Furthermore, walking, street, connectivity, and paths also showed some correlations, mostly related to roads. These word frequency statistics and correlations provided the basis for the selection of the indicators and grouping of the categories.

#### 4.3.2. Road Attributes

Within the dimension of road attributes, there are three categories: accessibility, street connectivity, and the street environment. Accessibility means the ease with which a place may be reached, while physical accessibility studies were founded on location theory [44]. Travel time and distance are considered to be the two important factors influencing physical accessibility, and they can be improved or limited by transportation [45]. For distance or proximity, common criteria used to examine are Euclidean distance, Manhattan distance, and network distance [45,46]. Travelling distance has a limiting effect on park visits, with the maximum park visitation decreasing as the travelling distance increases. The accessible distance and maximum travelling distance varied by the mode of travel and the frequency of park visits. [47,48]. Tu et al. [48] studied four cutoff points, namely 1 km, 2 km, 5 km, and 10 km. More than half of the people who decided to walk chose a park within 1 km. More than 95% of people who used parks frequently chose parks within 5 km from their homes. On the basis of the frequency of use, Liu et al. [49] classified residents as infrequent users, moderate users, and frequent park users. For moderate park users, the existence of parks within a walking distance of 500 m was very important. For frequent park users, a distance within 1000 m was better. Priess et al. [50] discovered that walking is the most common way to visit parks and the maximum limit of walking distance is 900 m, which equivalent to 15 min of walking time. The second indicator, along with distance, is travel time. In terms of urban-level connectivity assessment, Stoia et al. [51] and Iojă et al. [52] also noted that the immediate vicinity is about 50 m, 5 min of walking time corresponded to approximately 300 m, and 10 min of walking time was about 500 m. The 15 min accessibility has also become a time threshold for the accessibility of urban public service facilities, including urban parks [53,54]. Regarding accessibility, many studies have mentioned that walking is the most important way for people to visit parks. For large parks, the connectedness is affected by the amount of public transportation [41,45] and the number of bus stops [47,55]. The ease of parking for private vehicles is also a significant factor in the frequency of park visits [15,18].

A street network is designed to connect spatially separated places and enables people to move from one place to another. Street connectivity has important effects on the accessibility of potential destinations, travel choices, and quality of life [56]. He et al. [28], Tao et al. [2], and Sugiyama et al. [57] all identified intersection density or the number of intersections within the buffer zone as the indicator of street connectivity.

According to the review of the selected articles, many scholars mentioned that the street environment plays an important role in road networks, especially in the walking environment. Five aspects have been used to evaluate the street environment: segment type, street facilities, street slope and pavement quality, street greenery and shade, and the street signage system. For segment type, Wimbardana et al. [58] proposed low-volume roads, high-volume roads, pathways, sidewalks, pedestrian streets, and footpaths. People prefer the sidewalks and pedestrian streets because cars are prohibited, and high-volume roads make crossing the street difficult. Street facilities can greatly encourage walking if they are well maintained and the width of the sidewalk is suitable [59]. Common facilities include trash bins, seating furniture, business settings and toilets, and entrance gates. To evaluate the completeness of street facilities, researchers usually used a dummy variable (presence or absence of a facility) or counted the total number of street facilities. Regarding the street slope and pavement quality, the steepness of a street had an impact on the accessibility of the walkway or public space [60]. Researchers have used relatively subjective opinions for the standard of steepness. Rigolon et al. [61] noted that a flat surface was less than 3%, a slight hill was an estimated slope of 3–10%, and a steep hill was an estimated slope of more than 10%. However, Wimbardana et al. [58] classified steepness with thresholds of 12% and 25% for flat, slight hills, and steep hills. The quality of the paved areas is also important, especially for pedestrians, including the materials (concrete, gravel, or dirt) covering the path, and the absence or presence of bumps, cracks, holes, and street obstacles (poles, trees) [61]. For the road per se, a street with insufficient width can cause congestion. Street greenery and shade boost the value of the road from both aesthetic and practical perspectives. Having canopies and shade in summer is important, which can increase urban walkability, encourage usage of the urban environment, improve thermal comfort, and reduce the risk of sun exposure [62]. Street greenery can be measured by the type and volume of vegetation in a photo, or by counting the trees, shrubs, grass, and flowers [28]. Regarding the street signage system, Zhang et al. [17] and Rosli et al. [63] believed that signage systems and information boards can make it easy for people to visit their destination, avoiding the wrong route and saving travel time. The results are shown in Table 4.

#### 4.3.3. Park Attributes

There are three categories under the dimension of urban park quality, which are the spatial scale, facilities and amenities, and natural elements, as shown in Table 5.

Three indicators were mentioned in the category of spatial scale, namely the availability of green space, the park area, and green space per capita. First, the availability of green space means that there is available green space around “me” [64]. The second is the park area (with the service radius). Fan et al. [45] stated that a park’s area has been regarded as one of the most important indicators of the quality of green space. According to a study by Zhai et al. [65], different countries have different park services and park areas. In the US, a neighborhood park is more than 6 hectares (ha) (with a 400–800 m service radius). In Japan, a neighborhood park is more than 2 ha (with a 500 m service radius). In China, a community park should be greater than 1 ha (with a 500–1000 m service radius). He et al. [28] noted that park area was measured by the total area of parks within a neighborhood buffer, within 800 m or 10 min walking distance. In terms of public green space area per capita, Dong et al. [16] mentioned that this could be obtained from the total public green space area divided by the number of residents. We can conclude that the three indicators are layered progressively. The first layer is the availability of green space. The second is green space that is not only available but also has a reasonable size. The third layer is the per capita area after taking the population data into account.

Through word density analysis and clustering of the total indicators, facilities and amenities were the most frequently mentioned words. These affect users’ activity and the park’s quality, including (1) park infrastructure facilities, including lighting, seating furniture [41], public toilet [4], and trash bins [18]; (2) park recreational amenities [41], such as sport/fitness facilities [17,66] and children’s entertainment facilities [17,18,42,43]; (3) cultural and aesthetic facilities, such as landscape sketches and sculptures [1,15,43].

To be close to nature for relaxation is the main reason why people visit urban parks; therefore, the natural element is a significant factor that affects park users’ behavior [1,6]. Four indicators were mentioned many times by scholars, namely, vegetation, shade trees, water bodies, and animals. For vegetation, the most common measurement is vegetation quality or vegetation coverage (measured using the normalized difference vegetation index). From the users’ perspective, shade trees can provide a nice cool space, especially in summer. The advantages of water bodies and opportunities for animal encounters were also revealed by Park [6], Zhang et al. [15], Veinberga and Zigmunde [67], and Zhang et al. [17].

#### 4.3.4. Perception Attributes

The perception of urban parks is an emotional connection between people and places [68]. After a comparative analysis of the indicators, the indicators of perception attributes are shown in Table 6.

Perceived accessibility, distinct from physical accessibility, refers to individuals’ subjective assessment of the actual distance or the time needed to cover the distance [69]. The perceived proximity or convenience has shown more importance than physical accessibility [6,69] and is more likely to motivate the use of green space [66]. Perceived accessibility has better explanatory and predictive power than physical accessibility. Park planners should consider people’s perception and preferences to facilitate the development of urban parks, for example, by making them convenient and walkable.

In addition to perceived proximity, perceived safety is another factor that influences the usage of parks. For example, McCormack et al. [70] stated that homeless people, drug addicts, a lack of security, and secluded paths and areas make people feel unsafe and impede people from visiting the parks. Moreover, Chu et al. [71] stated that litter suggesting criminal incidents such as broken glass, empty cans, and drug needles can also make people feel unsafe. Bahriny and Bell [72] believed that park management is related to the park’s surfaces, vegetation, and sanitary conditions.

According to the systematic review, the indicator “aesthetic” was mentioned more than 10 times. First, discussions on aesthetics have mainly focused on naturalness, such as varied vegetation [15,41], grass, flowers, landscapes, and water bodies [70]. The second is attractiveness, including landscape design elements such as fountains and architecture, or cultural attractions. Studies have shown that attractiveness was a very important attribute of public space [73,74]. A highly attractive urban public space can affect public perception of distance, prompting people to ignore the actual distance within a certain range and enhance recreational walking and physical activities [74]. The last is cleanliness, which is very basic for a public space.

In Kaplan’s perceptual model, coherence, legibility, complexity, and mystery are important predictors of perceived preferences. Shayestefar et al. [75] explored Kaplan’s preference matrix in the assessment of urban parks and visual attributes. In the terms of coherence, it is related to the organization of elements, the order of scenes, and the degree of material uniformity [67]. The meaning of place and the presence of harmony can be improved by the extent of repetition and sequences [76,77]. Legibility represents uniqueness, allowing people to distinguish it from other places through distinctive features and landmarks. Road information legibility refers to the ability to quickly find relevant information during wayfinding. It is about being safe, not getting lost, and exploring the way. Complexity can be understood as diversity and variety. Appropriate complexity can lengthen people’s usage time and maintain interest. Mystery means that some information is invisible, hiding part of the landscape, and stimulating people’s curiosity to explore. It can be measured by the permeability of the enclosure, such as visual access and physical access [75]. Both visual open and physical open have an impact on the connectedness among urban parks. It can be seen that good visual attributes and understanding users’ perceived preferences are very important for the assessment of park environments.

#### 4.3.5. Individual Attributes

**Figure 6:**
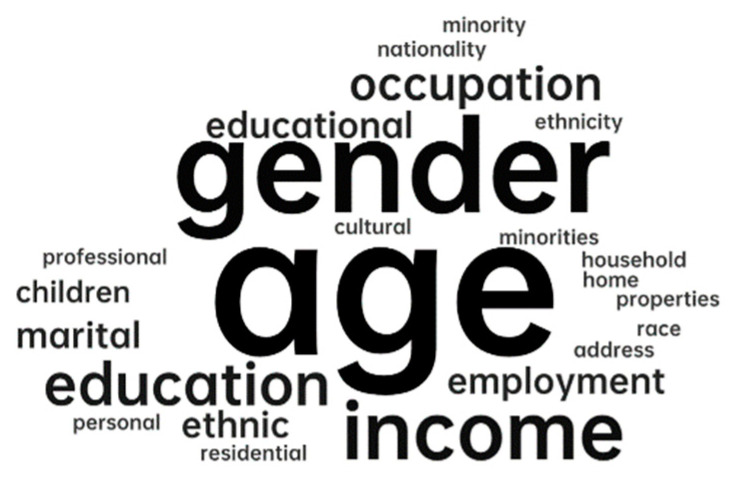
Word density cloud of sociodemographic characteristics.

This research studied the connectedness among parks from the users’ perspective. Considering the perception of participation, it is worth discussing whether the personal characteristics of the participants will affect the differences in perception. This part analyzes the sociodemographic characteristics of the population. The indicators are shown in Table 7. Among the 54 articles, 83% of the studies focused on all ages of the population. Only 11% of the studies focused on specific age groups, mainly children and the elderly. In addition, nearly half (43%) of the studies identified sociological demographic information as a variable affecting people’s demand for park quality and roads. According to the analysis of word frequency density, as shown in Figure 6, the most frequently mentioned variables were age, gender, income, education, and employment or occupation.

In addition to the sociodemographic characteristics, motivation also determines the users’ visits and whether they cross different parks. Sukwai et al. [78] argued that people are willing to go further for specific parks that are filled with activities they enjoy or have the attributes of interesting physical activity. It has also been suggested that if the purpose of visiting a park is to engage in physical exercise, then distance may be less likely to be a predictor of choices [29]. Therefore, the motivation and activity type of users are also key factors. According to the different needs of the users, necessary activities refer to outdoor activities that have to occur; spontaneous activities involve the individual’s autonomous behavior, such as walking and exercising; social activities emphasize the need for interpersonal communication [79]. According to this classification standard, the motivation for visiting the parks can also be divided into three types: necessity, spontaneity, and sociality.

**Figure 7:**
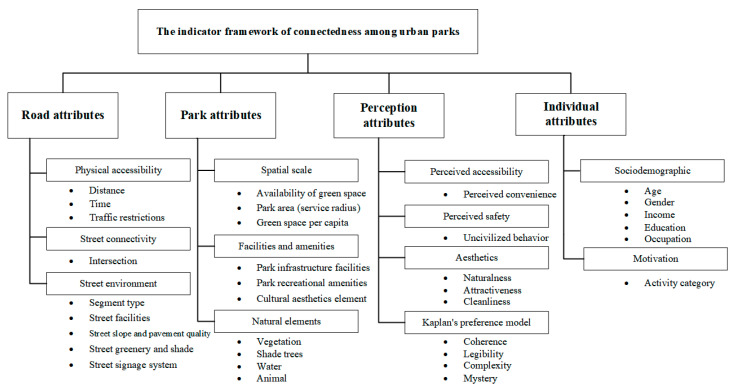
An indicator framework for connectedness among urban parks.

In summary, an indicator framework for connectedness among urban parks was developed, which included 4 dimensions, 12 categories, and 34 indicators, as shown in Figure 7.

## 5. Discussion

### 5.1. Connectedness Paths: Tangible and Intangible

**Figure 8:**
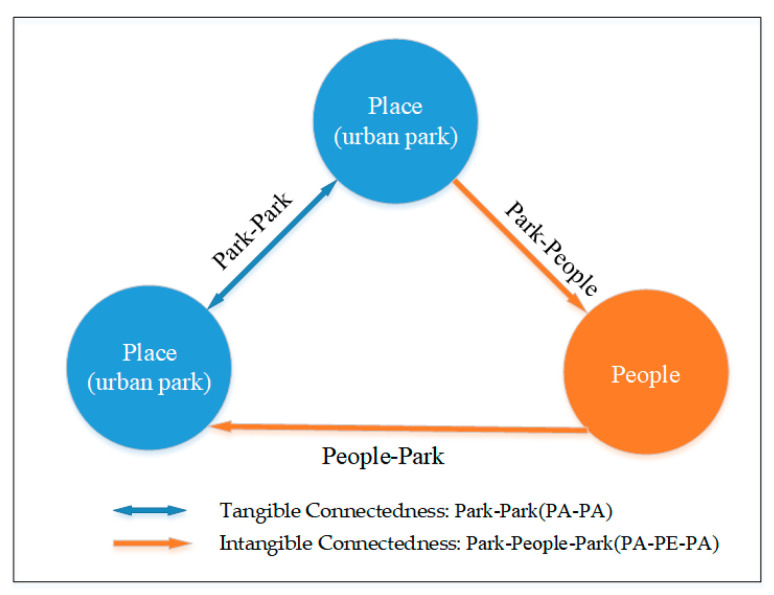
Relationships between two connectedness paths among urban parks.

Through the in-depth reading of the articles and construction of the indicator framework, we found that there are two different paths of connectedness among urban parks: Park–park (PA–PA) and park–people–park (PA–PE–PA), as shown in Figure 8.

The park–park (PA–PA) connectedness path, which refers to the connectedness of the road network, involves research into the road attributes between two physical spaces. This connectedness is tangible and explicit, such as roads, rivers, and walking systems. From the users’ perspective, road attributes affect people’s access to green space [61], e.g., accessibility [43] and street connectivity [57]. Dong et al. [16] argued that for the continuity of the park experience, the residents’ access to a certain green space (related to accessibility) was the first step, and movement among green spaces (related to connectivity) was the second step. Niță et al. [80] took the quantity, quality, and connectedness of neighborhood landscapes into consideration, and mentioned that neighborhood green spaces need to be better connected to public infrastructure, such as bike paths, greenways, and other city parks. Trancik [81] declared that linkage is the glue of the city and that a system of connections or networks can be developed by streets, pedestrian ways, linear open spaces, or other linking elements that physically connect the parts of the city. Thwaites et al. [21] believed that streets and their capacity to connect a diversity of parks may have the potential for regeneration and rejuvenation. Thus, the PA–PA path primarily focuses on the impact of road attributes on urban parks.

The park–people–park (PA–PE–PA) connectedness path focuses on the people’s perception of park attributes, taking the park users as the mediator. Users in this path establish an intangible and indirect connection between urban parks. In other words, this concept explores park quality and the sense of place through users’ activities and perceptions. Norberg-Schulz [82], on the basis of Forman’s corridor–patch–matrix structure, explored the relationship between human function and spatial expression and suggested that human beings achieve spatial connectivity through the development of and participation in the physical environment [21]. There was intangible connectedness between green spaces, for example, the urban space axis [83], the time axis, the cultural axis [84], the landscape axis, and visual corridors [85]. This indirect connectedness can be realized through users’ perception. Humans have the ability to sense visual coherence and organization in urban parks and stimulate and sustain sequential experience [86]. Aesthetic and psychological associations, such as the continuous output of culture in proximity, can effectively offer a continuous experience. Chu et al. [71] mentioned that improvements in park quality can effectively encourage residents to visit again, and several parks with high correlations can generate group effects in attracting residents. Wan et al. [1] also declared that psychological factors played a potential mediating role in the association between physical factors and the relationship between people and the environment. In short, the PA–PE–PA path concentrates on the park attributes, that is, improvements in the park’s quality trigger users to visit and establish indirect connectedness through their perceptions.

Some studies have focused on the tangible connectedness between urban roads, such as urban greenways [87,88], street connectivity [89], and walkability [90]. However, the indirect and intangible connectedness between cultural elements, the continuity of visual information, and the design of landscape axes between urban parks cannot be ignored. The two connectedness paths are not separate. Parks are discrete objects within a larger system of urban amenities. They have mosaic-like characteristics, which determine not only the attention paid to the park mosaics but also the roads connecting them. Better coordination between the road environment and the park quality can effectively enhance the range of residents’ activities and increase the vitality of the city.

### 5.2. Physical Connectedness and Perceived Connectedness: Objectivity and Perceptions

After aggregating the four dimensions, we found that road attributes and park attributes were based on the physical place and have been analyzed in terms of many objective environmental elements. They can be measured objectively and can be summarized as physical connectedness. This connectedness includes physical accessibility, street connectivity, the street environment, the spatial scale, facilities and amenities, and natural elements. These indicators of physical connectedness influence the environmental, aesthetic, and recreational benefits that urban parks provide to their users [91]. They also determine people’s access to the parks and the connectedness between urban parks. However, the realization of these benefits is influenced not only by the inherent characteristics of physical connectedness but also by how people perceive those characteristics.

Perceived connectedness is based on people and mainly concentrates on the subjective perception of physical connectedness, including perceived accessibility and perceived safety. In addition, it also includes some psychological and perceptual preferences, such as aesthetic dimensions and Kaplan’s preference model. An understanding of perceived connectedness can reveal the main concerns and needs of users in urban parks, which can help to clarify the priorities and focus of urban construction and planning. As the subject of perception, people’s social demographic characteristics and activity motivations will also affect the result of their perception.

**Figure 9:**
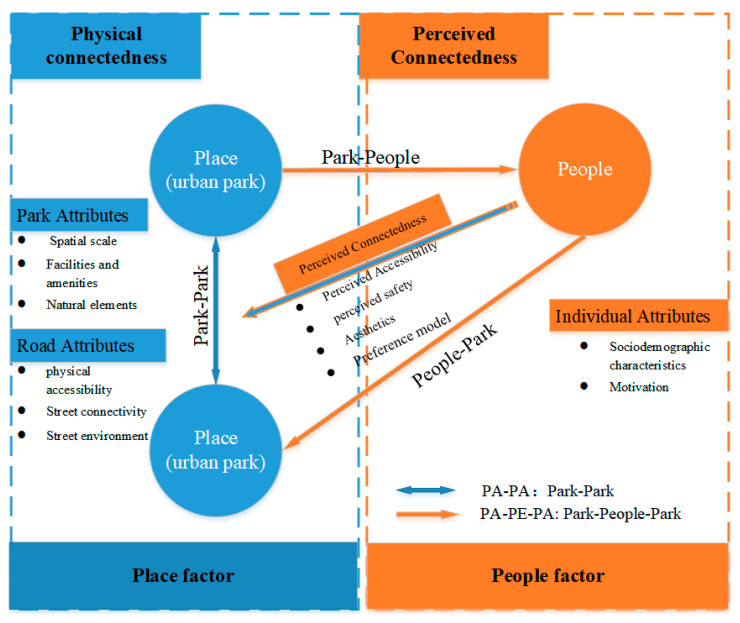
The relationship between physical and perceived connectedness.

Physical connectedness and perceived connectedness are interrelated and complementary (see Figure 9). Physical connectedness (road attributes and park attributes) is a prerequisite for urban connectedness, which affects and determines users’ willingness to visit. Perceived connectedness, namely users’ participation and perception, facilitates the connection between parks and people, completing the process of attachment to place.

We have discussed the relationship between parks, namely two different connectedness paths, PA–PA (road attributes) and PA–PE–PA (park attributes), and also analyzed the relationship between people and parks, physical connectedness and perceived connectedness, as well as users’ perception of the park, road, and physical connectedness. The connection between people seems to be related to the two relationships mentioned above. The use and connection of public space can affect people’s place attachment to the space and the possibility of more contact with other people [92,93]. For example, various elements and facility arrangements in the open space can attract people together, resulting in more social cohesion [93,94]. In order to strengthen social interaction and social cohesion between people, it is important to consider not only good physical access and welcoming spaces, but also appropriate management and spatial configuration, encouraging different groups and people to share these spaces [93]. These open green spaces serve as places of potential social interaction, where weak, one-off interactions, as well as strong and more structured interactions, can occur [94]. In short, by using multiple parks in a city, people can connect to certain places as well as other people who use them, which can lead to social cohesion [95]. Therefore, we can believe that the connection between parks and the connection between people and parks will enhance the social interaction between people and strengthen social cohesion to some extent.

### 5.3. Recommendations for Future Research

This study filled out the research into physical and perceived connectedness among urban parks and users’ visits by using a systematic literature review. For future research directions, several aspects can be considered. First, the identified research gaps show a lack of studies from most of the global south; future studies can focus on the parks’ connectedness in Africa, South America, and Australia, which will replenish the current knowledge about the connectedness among urban parks globally. Second, empirical evaluations of the framework can be carried out. In this aspect, all indicators can be weighted by means of the analytic hierarchy process (AHP) or expert scoring methods to determine the level of importance of each indicator. The framework can then be used to measure the degree of connectedness in specific cities. According to different levels of connectedness, reasonable suggestions should be proposed. Third, on the basis of physical connectedness and perceived connectedness, it is worth discussing residents’ attitudes toward connectedness in the future. For example, studies could explore whether physical connectedness or perceived connectedness is more important to residents, and whether these conclusions differ depending on age and gender. This study also mentions the different paths of connectedness, and further studies can be carried out on these, such as whether, for residents, the road attributes or the park attributes are more conducive to promoting people to go there. A more accurate conclusion can be obtained through a series of correlation analyses and regression analyses. Lastly, urban parks are not only for people but also for flora and fauna. For different groups benefiting from urban parks, future research can consider the combination of user-based social function connectedness and biodiversity-based ecological connectivity, integrating multiple interests and realizing the unity between ecology and society.

### 5.4. Theoretical and Practical Implications, and Limitations

Our theoretical contribution is the introduction of a connectedness framework for urban parks that reveals the integrated connections between places and people. Based on the existing linkage theory, the research perspective and scope were enriched, the theory was expanded from physical connectedness to perceived connectedness, and the general knowledge on connectedness was integrated and summarized. In terms of practical applications, these frequently mentioned indicators can play a guiding role for urban planners to help them renew and update urban green spaces, from planning and improving roads to the optimization and renovation of park quality.

This study had certain limitations. First, the definition of the search terms (for the systematic literature review) was obtained after repeated discussions among the three authors, but it was found that some omissions were inevitable in the later part of the process. However, according to saturation theory, the authors ensured that the indicators that appeared in the chosen articles were included in the set of all raw extracted indicators to avoid deviations in the indicators caused by differences in terminology. In order to ensure the objectivity and fairness of the process of selecting the indicators, the Delphi method, expert scoring, or surveys could be adopted in the future to verify the objectivity and saturation of the indicators. Second, the systematic literature review involved many countries, but the planning standards and cultural backgrounds of parks in different countries are different, such as park service radius, and, thus, the details of indicators can be improved later according to a specific country or city.

## 6. Conclusions

The purpose of this study was to explore the connectedness among urban parks. The first major finding (answering sub-RO1) indicated that the majority of the case studies in the selected articles were from Asia, Europe, and North America, while countries from other continents were not covered. In terms of the methodology, quantitative research dominated the research methods, accounting for up to 70%. As for the connectedness measurement methods, perceived connectedness has become more important, almost as much as objective connectedness measurement. An increasing number of scholars have begun to realize the combination of objective measurements and perceived measurements. According to the selection and organization of the indicators, a connectedness framework among urban parks was developed, which contained 4 dimensions, 12 categories, and 34 indicators (answering sub-RO2). Finally, responding to RO3, this study discovered paths of connectedness based on the framework of the indicators. One is park–park (PA–PA) connectedness, which refers to tangible and direct connections, focusing on the road attributes. The other path is park–people–park (PA–PE–PA), which refers to intangible indirect connections, concentrating on the park attributes. The user is the intermediate element, and the connectedness between the urban parks is realized through the visits of the users. In addition, compared with previous studies, which only focused on physical space, this study proposed the integration of perceived connectedness and physical connectedness, not only emphasizing the importance of physical objective factors but also stressing users’ participation and perceptions. In this way, it promotes connectedness and movement between urban parks and people.

## Figures and Tables

**Table 1:** Key search query.

1st Group of Search Query	2nd Group of Search Query	3rd Group of Search Query
Connectedness	Park *	User
Connectivity	Urban Park *	Resident *
Continuity	Public green space *	Tourist *
Coherence		Person *
Proximity		Individual
Accessibility		People
Walkability		Human being

Note. * = any group of characters, for finding words with any possible ending.

**Table 2:** Criteria and reasons for inclusion and exclusion.

Inclusion	Exclusion	Reason
2017–2022	Before 2017 (except for references and citations)	The search results showed that the number of articles has increased significantly since 2017
Articles, conference articles, reviews, conference reviews	Other types, for example, Books and chapters in books	The objective of an article is more focused; it is easier to obtain the full text
English language	Non-English	English-language articles are the most numerous in the database; it is a world language and easier to understand
Physical or social connectedness	Evaluations of ecology or landscape connectivity	The study does not discuss ecological connectivity but focuses on the users’ perspectives
Residents or tourists	The disabled, patients	To determine the mean and range of the indicators’ measurement items, such as walking distance and walking time, some populations were excluded for physical reasons.
Urban public green spaces or urban parks	Green buffers, urban forests, marshlands, habitats	The research scope refers to public green spaces for leisure and entertainment; other green spaces not open to the public were excluded

**Table 3:** The geographical distribution of the case study area.

Regions	Country	Total Number	(%)	City/Town
Asia		37	70%	
	China	20		Beijing, Shanghai, Wuhan, Changchun, Hongkong, Shenzhen, Taipei, Yangzhou, Nanjing, Ningbo
	Iran	5		Tehran, Gorgan
	Indonesia	2		Palembang, Jakarta
	Singapore	2		-
	Turkey	2		Trabzon
	Malaysia	1		Batu Gajah, Perak
	Bangladesh	1		Dhaka
	India	1		Kolkata
	Saudi Arabia	1		Dammam
	Thailand	1		Chiang Mai
	Vietnam	1		Da Nang
Europe		9	17%	
	Romania	3		Bucharest, Mehedinți County
	Portugal	2		Lisbon, Coimbra
	Latvia	1		-
	Germany	1		Leipzig
	Lithuania	1		Vilnius
	UK	1		Newcastle upon Tyne
North America		7	13%	
	USA	7		New York City, Minneapolis–Saint Paul, Denver, Los Angeles, Arizona’s metropolitan areas

**Table 4:** Indicators of road attributes.

Dimension	Category	Indicator	Sources (No. in Supplementary File S1)
Road attributes	Physical accessibility	Distance	2,5,7,9,11,14,15,20,25,28,34, 35,39,40,44,46,47,48,54
		Time	
		Traffic restrictions	
	Streetconnectivity	Intersection	8,28,30,39,51,52,53
	Street environment	Segment type	1,14,16,24,30,45,46,50,51
		Street facilities	
		Street slope and pavement quality	
		Street greenery and shade	
		Street signage system	

**Table 5:** Indicators of park attributes.

Dimension	Category	Indicator	Sources (No. in Supplementary File S1)
Park attributes	Spatial scale	Availability of green space	2,4,8,9,14,15,16,23,29,30,31, 35,36,44,46,47,52
		Park area (service radius)	
		Green space per capita	
	Facilities and amenities	park infrastructure facilities	1,3,5,11,14,15,16,19,20,24,25, 26,29,30,35,37,38,39,44,46
		park recreational amenities	
		cultural and aesthetics element	
	Natural elements	Vegetation	5,10,13,16,20,21,23,24,25,27, 29,32,34,36,43,44,46,47,50
		Shade trees	
		Water	
		Animals	

**Table 6:** Indicators of Perception attributes.

Dimension	Category	Indicator	Sources (No. in Supplementary File S1)
Perception attributes	Perceived accessibility	Perceived convenience	1,3,13,19,26,29,32,38,42,49
	Perceived safety	Uncivilized behavior	3,7,19,24,28,30,31,33,37, 38,43,44,51,52
	Aesthetics	Naturalness	3,5,12,15,19,21,24,29,30, 39,49,50,51,52,54
		Attractiveness	
		Cleanliness	
	Kaplan’s preference model	Coherence	6,7,21,22
		Legibility	
		Complexity	
		Mystery	

**Table 7:** Indicators of individual attributes.

Dimension	Category	Indicator	Sources (No. in Supplementary File S1)
Individual attributes	Sociodemographic characteristics	Age	1,3,4,5,6,8,9,10,11,15,17,19,23,26,28,30,31,32,33,34,41,42,50
		Gender	
		Income	
		Education	
		Occupation	
	Motivation	Activity category	23,24,30

## Data Availability

Not applicable.

## Supplementary Materials

The following supporting information can be downloaded at: https://www.mdpi.com/article/10.3390/ijerph20043652/s1, File S1: Key information statistics for 54 selected articles of the systematic literature review; File S2: Raw indicator details of 54 articles.
